# Unexpected diagnosis following screening breast ultrasound

**DOI:** 10.1002/ccr3.3014

**Published:** 2020-06-01

**Authors:** Federico Cammertoni, Piergiorgio Bruno, Natalia Pavone, Piero Farina, Christian Colizzi, Antonella Coli, Massimo Massetti

**Affiliations:** ^1^ Department of Cardiovascular Sciences Cardiac Surgery Unit Fondazione Policlinico Universitario “A. Gemelli” IRCCS Rome Italy; ^2^ Department of Pathological Anatomy Fondazione Policlinico Universitario “A. Gemelli” IRCCS Rome Italy

**Keywords:** cardiothoracic surgery, cardiovascular disorders, mediastinum, teratoma

## Abstract

Any instrumental examination may lead to unexpected diagnosis that in turn can radically change the clinical pathway of a patient.

## Graphical abstract

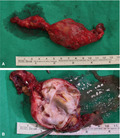

Breast ultrasound is of paramount importance for the prevention of breast cancer. In this report, we describe the unexpected finding of a mediastinal teratoma in a young, asymptomatic woman, who eventually required surgical excision.

A 43‐year‐old woman was referred to our hospital after a mediastinal mass was incidentally found on a breast ultrasound performed for screening purposes. Chest computed tomography, magnetic resonance imaging, and transoesophageal echocardiogram revealed a heterogeneous, 10 × 6 cm cystic mass with areas of septal calcifications in the anterior mediastinum (Figure 1A‐D). Although adherent to the pericardium and contiguous to the cavo‐atrial junction, there were no signs of invasiveness of the heart and great vessels. Surgical resection was accomplished through median sternotomy. The cystic mass had solid areas (Figure 2A,B) and was adherent to the pericardium medially, to the right lung laterally and inferiorly, to the cavo‐atrial junction posteriorly. Highly vascularized by the surrounding tissues, it was excised completely through blunt dissection. Histological examination (Figure 3) revealed residual thymic tissue. The cysts were lined by stratified or columnar ciliated epithelium. Neural tissue, fibrous tissue, and nests of cartilage were found. The final histopathological diagnosis was mature cystic teratoma. Teratomas are germ cell tumors that usually originates from gonads. Mediastinum is the second most common site of these heterogeneous neoplasms.^1^ Malignant transformation occurs in 10%‐20% of mediastinal teratomas.^2^ Regardless of their histopathology, surgical resection is the treatment of choice, especially when adhesions with the heart and great vessels exist.

**FIGURE 1:**
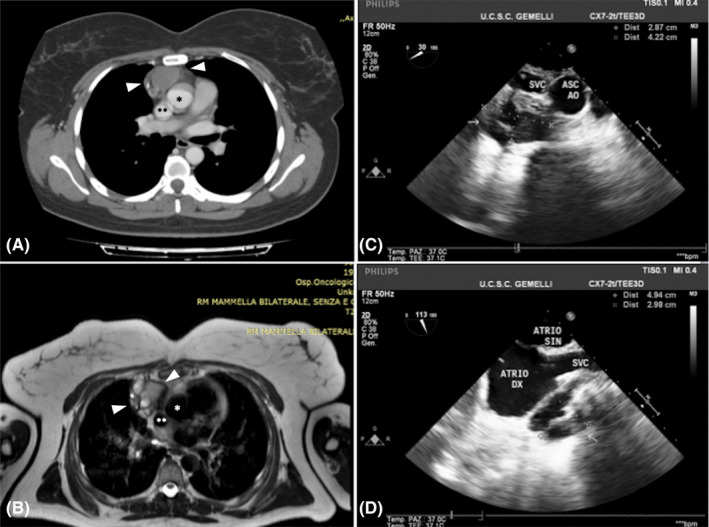
Chest computed tomography scan (A) and magnetic resonance imaging (B) showing the structure and the anatomical relations of the mediastinal mass (white pointers). In particular, the ascending aorta (*) and the superior vena cava (**) surround the posterior and medial margins of the mass. Transesophageal echocardiogram (C, D) confirms the strict adherence of the mass (arrows) to the superior vena cava (SVC), right atrium (ATRIO DX), and ascending aorta (ASC AO)

**FIGURE 2:**
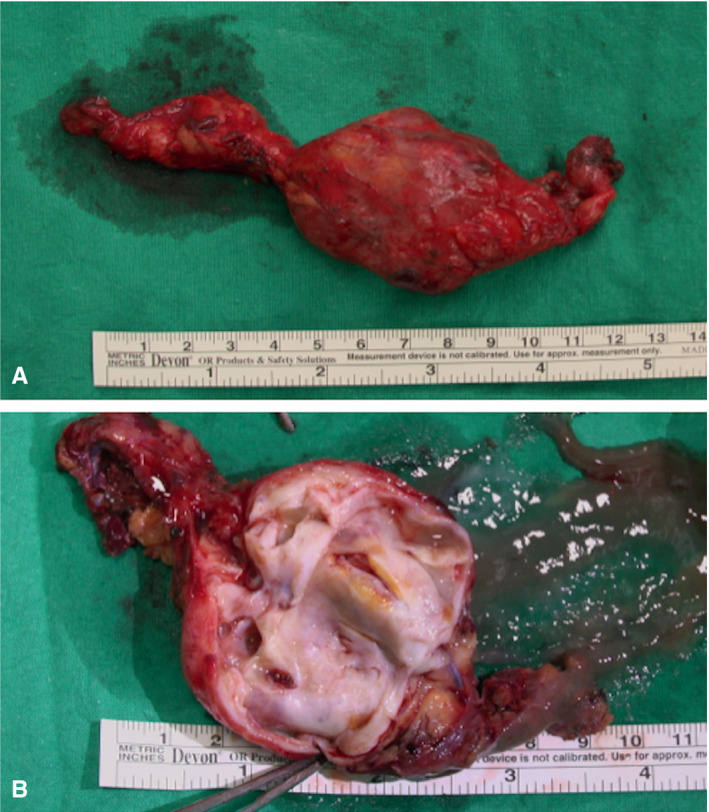
Intraoperative outer (A) and inner (B) view of the cystic, multiloculated mass. See text for details

**FIGURE 3:**
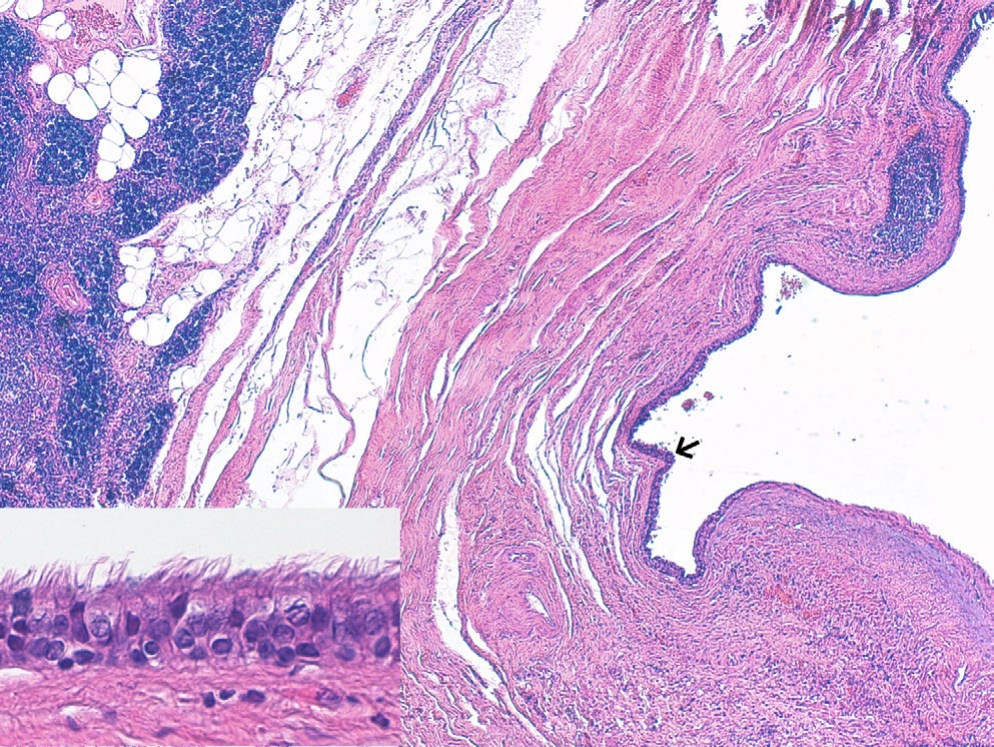
Histological analysis of the cystic teratoma. The cysts were lined by stratified or columnar ciliated epithelium (arrow) and other components as neural and fibrous tissue and nests of cartilage were found (inlet)

## CONFLICT OF INTEREST

Authors have no conflict of interest to disclose.

## AUTHOR CONTRIBUTIONS

FC and NP: wrote the manuscript. PF: proofread the manuscript and helped with English language editing. MM: performed surgery and supervised the scientific content of the paper. PB: performed surgery and supervised the scientific content of the paper. CC: performed echocardiography and collected related images. AC: performed histologic analysis and collected related images.

## References

[1] Mustafa OM , Mohammed SF , Aljubran A , Saleh WN . Immature mediastinal teratoma with unusual histopathology: a case report of multi‐lineage, somatic‐type malignant transformation and a review of the literature. Medicine (Baltimore). 2016;95(26):e3378. 27367976 10.1097/MD.0000000000003378 PMC4937890

[2] Motzer RJ , Amsterdam A , Prieto V , et al. Teratoma with malignant transformation: diverse malignant histologies arising in men with germ cell tumors. J Urol. 1998;159:133‐138. 9400455 10.1016/s0022-5347(01)64035-7

